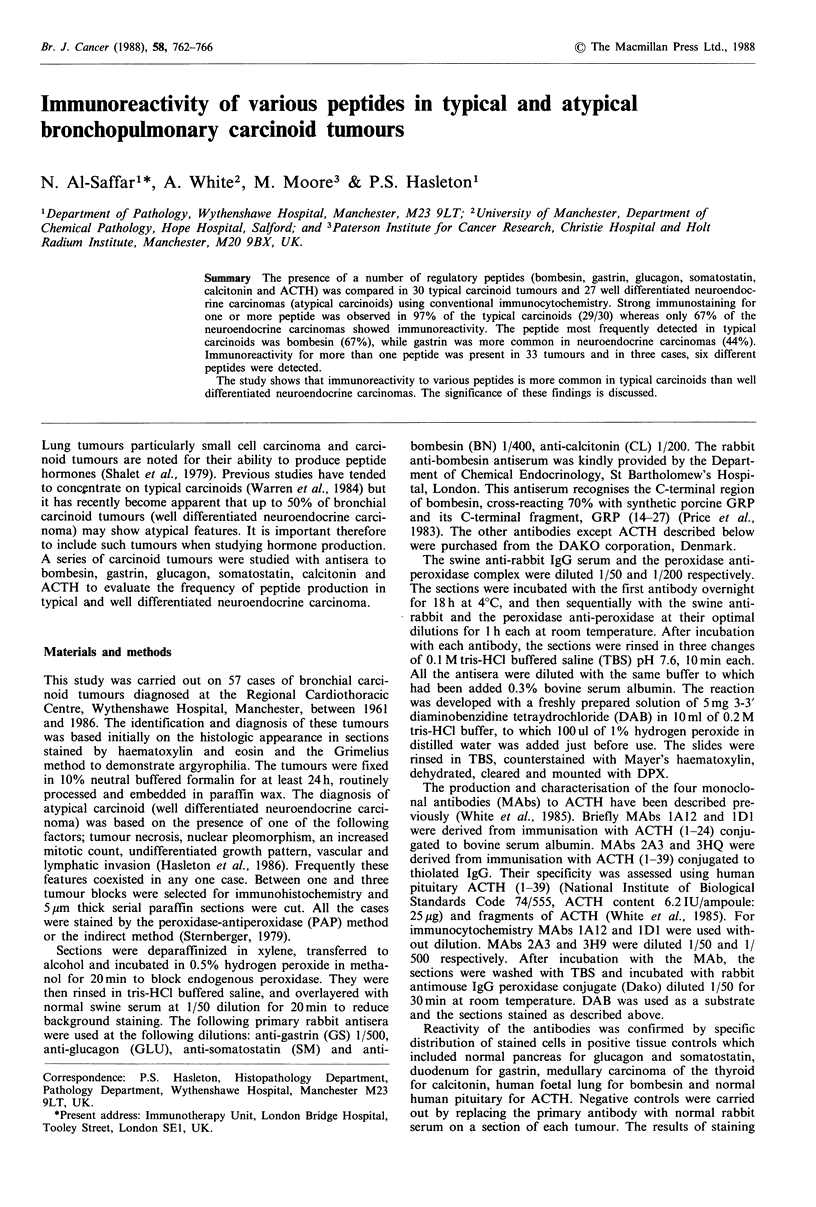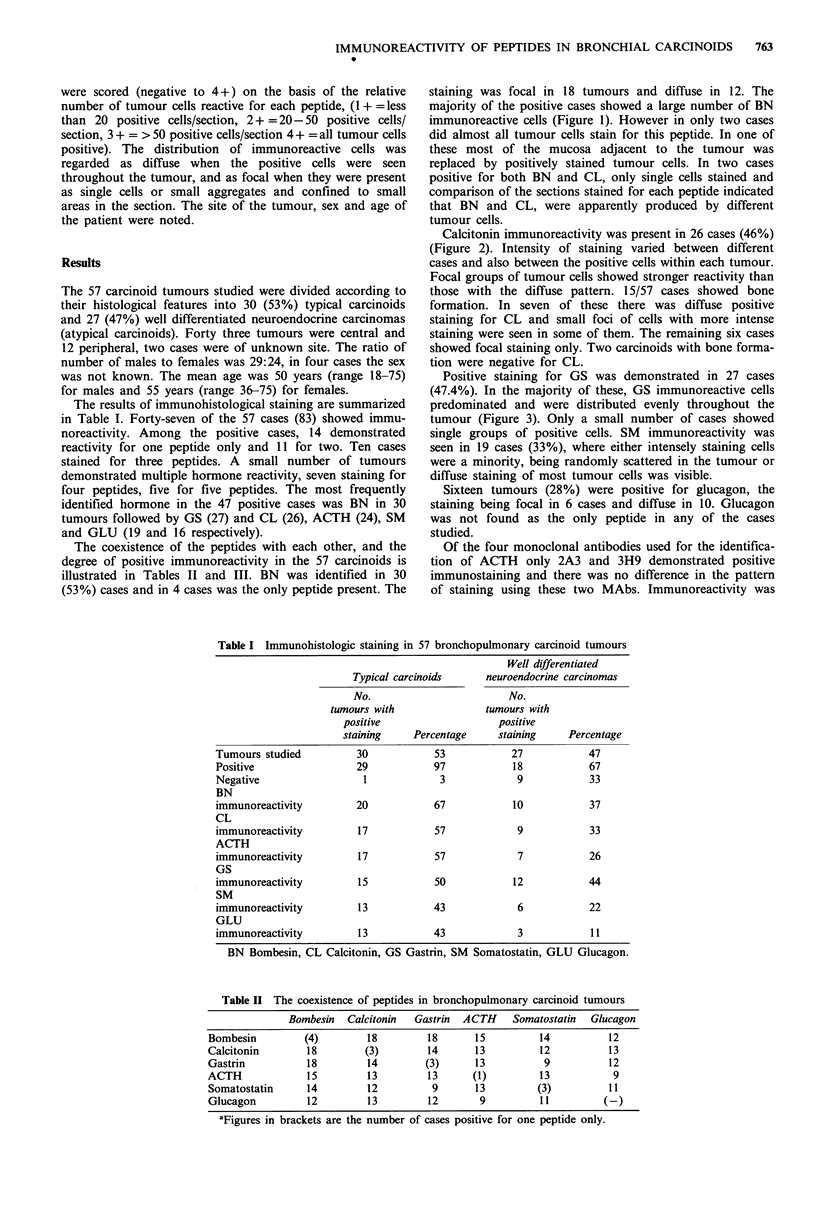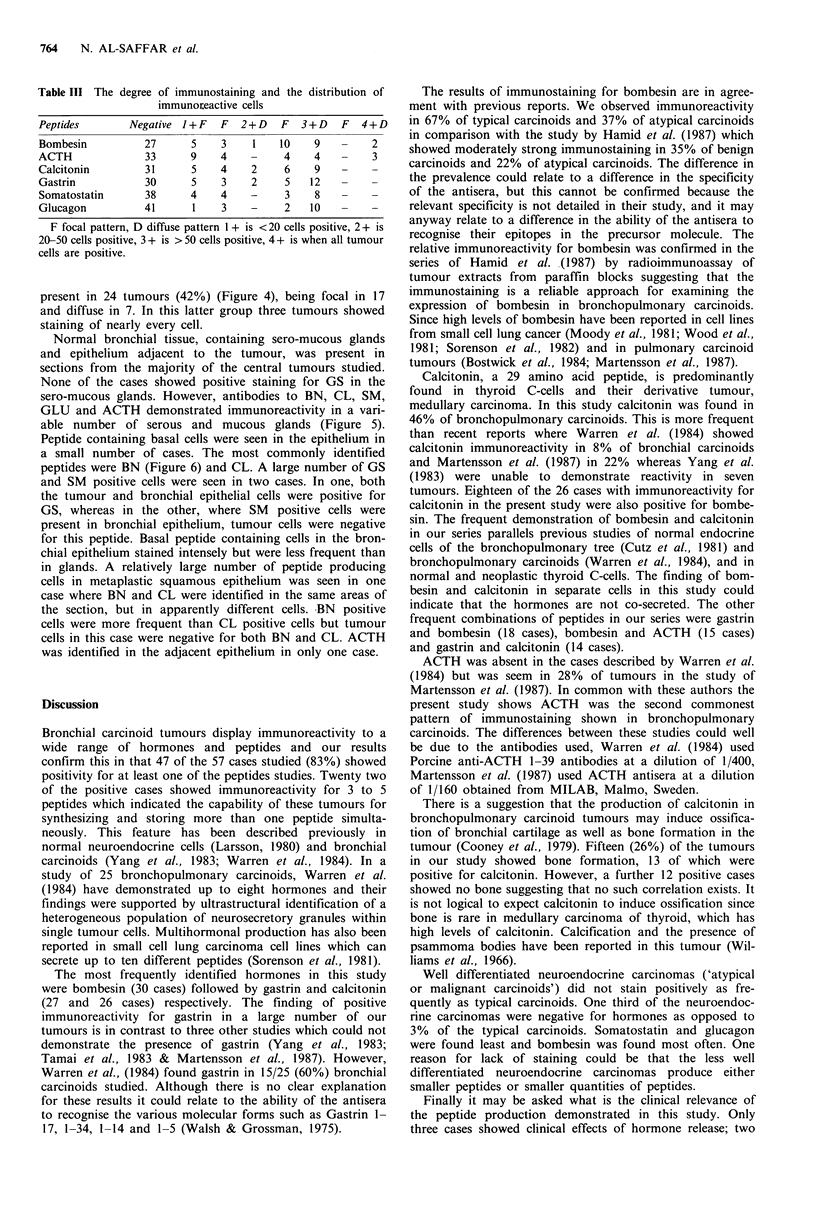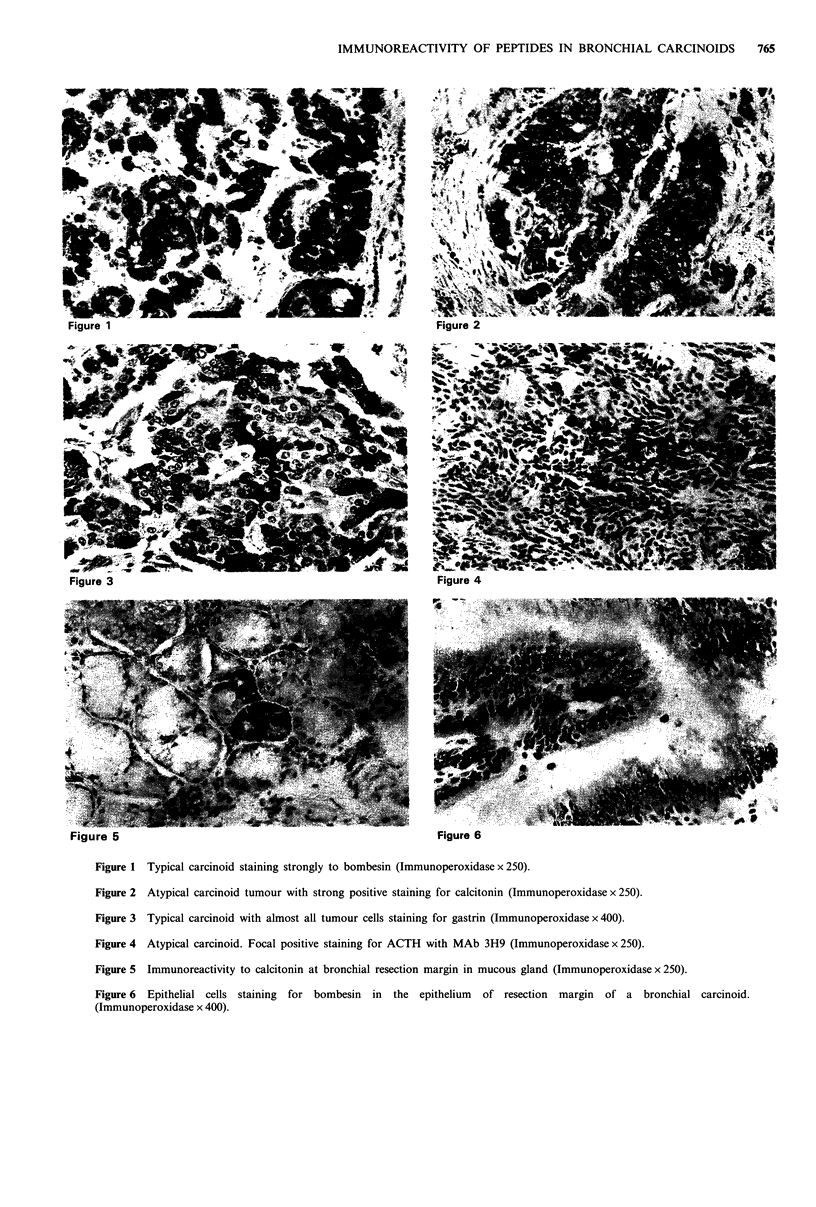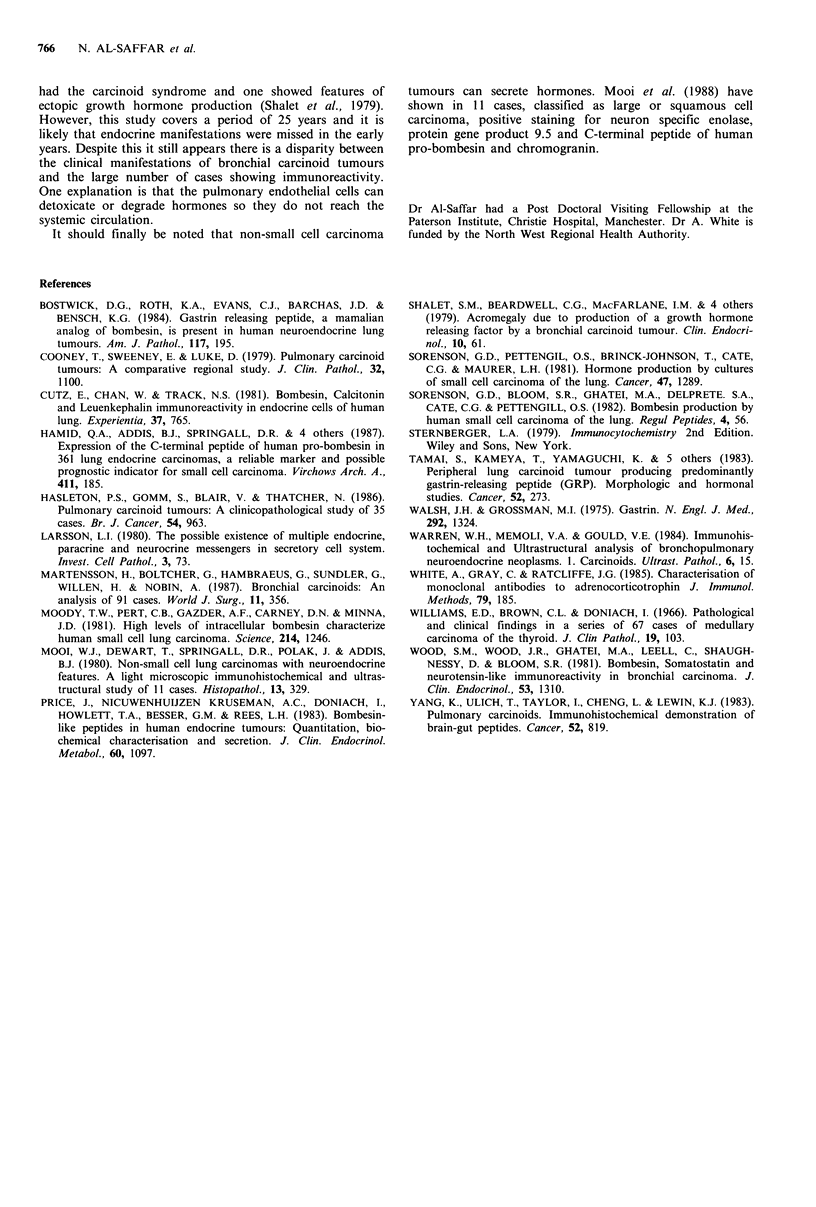# Immunoreactivity of various peptides in typical and atypical bronchopulmonary carcinoid tumours.

**DOI:** 10.1038/bjc.1988.304

**Published:** 1988-12

**Authors:** N. al-Saffar, A. White, M. Moore, P. S. Hasleton

**Affiliations:** Department of Pathology, Wythenshawe Hospital, Manchester, UK.

## Abstract

**Images:**


Br. J. Cancer (1988), 58, 762–766 © The Macmillan Press Ltd., 1988

### Immunoreactivity of various peptides in typical and atypical bronchopulmonary carcinoid tumours

N. Al-Saffar[1]*, A. White[2], M. Moore[3] & P.S. Hasleton[1]

[1]*Department of Pathology, Wythenshawe Hospital, Manchester, M23 9LT;* [2]*University of Manchester, Department of Chemical Pathology, Hope Hospital, Salford; and* [3]*Paterson Institute for Cancer Research, Christie Hospital and Holt Radium Institute, Manchester, M20 9BX, UK.*

**Summary** The presence of a number of regulatory peptides (bombesin, gastrin, glucagon, somatostatin, calcitonin and ACTH) was compared in 30 typical carcinoid tumours and 27 well differentiated neuroendocrine carcinomas (atypical carcinoids) using conventional immunocytochemistry. Strong immunostaining for one or more peptide was observed in 97% of the typical carcinoids (29/30) whereas only 67% of the neuroendocrine carcinomas showed immunoreactivity. The peptide most frequently detected in typical carcinoids was bombesin (67%), while gastrin was more common in neuroendocrine carcinomas (44%). Immunoreactivity for more than one peptide was present in 33 tumours and in three cases, six different peptides were detected.

The study shows that immunoreactivity to various peptides is more common in typical carcinoids than well differentiated neuroendocrine carcinomas. The significance of these findings is discussed.

Lung tumours particularly small cell carcinoma and carcinoid tumours are noted for their ability to produce peptide hormones (Shalet *et al.*, 1979). Previous studies have tended to concentrate on typical carcinoids (Warren *et al.*, 1984) but it has recently become apparent that up to 50% of bronchial carcinoid tumours (well differentiated neuroendocrine carcinoma) may show atypical features. It is important therefore to include such tumours when studying hormone production. A series of carcinoid tumours were studied with antisera to bombesin, gastrin, glucagon, somatostatin, calcitonin and ACTH to evaluate the frequency of peptide production in typical and well differentiated neuroendocrine carcinoma.

### Materials and methods

This study was carried out on 57 cases of bronchial carcinoid tumours diagnosed at the Regional Cardiothoracic Centre, Wythenshawe Hospital, Manchester, between 1961 and 1986. The identification and diagnosis of these tumours was based initially on the histologic appearance in sections stained by haematoxylin and eosin and the Grimelius method to demonstrate argyrophilia. The tumours were fixed in 10% neutral buffered formalin for at least 24 h, routinely processed and embedded in paraffin wax. The diagnosis of atypical carcinoid (well differentiated neuroendocrine carcinoma) was based on the presence of one of the following factors; tumour necrosis, nuclear pleomorphism, an increased mitotic count, undifferentiated growth pattern, vascular and lymphatic invasion (Hasleton *et al.*, 1986). Frequently these features coexisted in any one case. Between one and three tumour blocks were selected for immunohistochemistry and 5 $\mu$m thick serial paraffin sections were cut. All the cases were stained by the peroxidase-antiperoxidase (PAP) method or the indirect method (Sternberger, 1979).

Sections were deparaffinized in xylene, transferred to alcohol and incubated in 0.5% hydrogen peroxide in methanol for 20 min to block endogenous peroxidase. They were then rinsed in tris-HCl buffered saline, and overlayered with normal swine serum at 1/50 dilution for 20 min to reduce background staining. The following primary rabbit antisera were used at the following dilutions: anti-gastrin (GS) 1/500, anti-glucagon (GLU), anti-somatostatin (SM) and anti-bombesin (BN) 1/400, anti-calcitonin (CL) 1/200. The rabbit anti-bombesin antiserum was kindly provided by the Department of Chemical Endocrinology, St Bartholomew's Hospital, London. This antiserum recognises the C-terminal region of bombesin, cross-reacting 70% with synthetic porcine GRP and its C-terminal fragment, GRP (14–27) (Price *et al.*, 1983). The other antibodies except ACTH described below were purchased from the DAKO corporation, Denmark.

The swine anti-rabbit IgG serum and the peroxidase anti-peroxidase complex were diluted 1/50 and 1/200 respectively. The sections were incubated with the first antibody overnight for 18 h at 4°C, and then sequentially with the swine anti-rabbit and the peroxidase anti-peroxidase at their optimal dilutions for 1 h each at room temperature. After incubation with each antibody, the sections were rinsed in three changes of 0.1 M tris-HCl buffered saline (TBS) pH 7.6, 10 min each. All the antisera were diluted with the same buffer to which had been added 0.3% bovine serum albumin. The reaction was developed with a freshly prepared solution of 5 mg 3-3′ diaminobenzidine tetraydrochloride (DAB) in 10 ml of 0.2 M tris-HCl buffer, to which 100 ul of 1% hydrogen peroxide in distilled water was added just before use. The slides were rinsed in TBS, counterstained with Mayer's haematoxylin, dehydrated, cleared and mounted with DPX.

The production and characterisation of the four monoclonal antibodies (MAbs) to ACTH have been described previously (White *et al.*, 1985). Briefly MAbs 1A12 and 1D1 were derived from immunisation with ACTH (1–24) conjugated to bovine serum albumin. MAbs 2A3 and 3HQ were derived from immunisation with ACTH (1–39) conjugated to thiolated IgG. Their specificity was assessed using human pituitary ACTH (1–39) (National Institute of Biological Standards Code 74/555, ACTH content 6.2 IU/ampoule: 25 $\mu$g) and fragments of ACTH (White *et al.*, 1985). For immunocytochemistry MAbs 1A12 and 1D1 were used without dilution. MAbs 2A3 and 3H9 were diluted 1/50 and 1/500 respectively. After incubation with the MAb, the sections were washed with TBS and incubated with rabbit antimouse IgG peroxidase conjugate (Dako) diluted 1/50 for 30 min at room temperature. DAB was used as a substrate and the sections stained as described above.

Reactivity of the antibodies was confirmed by specific distribution of stained cells in positive tissue controls which included normal pancreas for glucagon and somatostatin, duodenum for gastrin, medullary carcinoma of the thyroid for calcitonin, human foetal lung for bombesin and normal human pituitary for ACTH. Negative controls were carried out by replacing the primary antibody with normal rabbit serum on a section of each tumour. The results of staining

Correspondence: P.S. Hasleton, Histopathology Department, Pathology Department, Wythenshawe Hospital, Manchester M23 9LT, UK.

*Present address: Immunotherapy Unit, London Bridge Hospital, Tooley Street, London SE1, UK.

were scored (negative to 4+) on the basis of the relative number of tumour cells reactive for each peptide, (1+ = less than 20 positive cells/section, 2+ = 20–50 positive cells/section, 3+ = >50 positive cells/section 4+ = all tumour cells positive). The distribution of immunoreactive cells was regarded as diffuse when the positive cells were seen throughout the tumour, and as focal when they were present as single cells or small aggregates and confined to small areas in the section. The site of the tumour, sex and age of the patient were noted.

### Results

The 57 carcinoid tumours studied were divided according to their histological features into 30 (53%) typical carcinoids and 27 (47%) well differentiated neuroendocrine carcinomas (atypical carcinoids). Forty three tumours were central and 12 peripheral, two cases were of unknown site. The ratio of number of males to females was 29:24, in four cases the sex was not known. The mean age was 50 years (range 18–75) for males and 55 years (range 36–75) for females.

The results of immunohistological staining are summarized in Table I. Forty-seven of the 57 cases (83) showed immunoreactivity. Among the positive cases, 14 demonstrated reactivity for one peptide only and 11 for two. Ten cases stained for three peptides. A small number of tumours demonstrated multiple hormone reactivity, seven staining for four peptides, five for five peptides. The most frequently identified hormone in the 47 positive cases was BN in 30 tumours followed by GS (27) and CL (26), ACTH (24), SM and GLU (19 and 16 respectively).

The coexistence of the peptides with each other, and the degree of positive immunoreactivity in the 57 carcinoids is illustrated in Tables II and III. BN was identified in 30 (53%) cases and in 4 cases was the only peptide present. The staining was focal in 18 tumours and diffuse in 12. The majority of the positive cases showed a large number of BN immunoreactive cells (Figure 1). However in only two cases did almost all tumour cells stain for this peptide. In one of these most of the mucosa adjacent to the tumour was replaced by positively stained tumour cells. In two cases positive for both BN and CL, only single cells stained and comparison of the sections stained for each peptide indicated that BN and CL, were apparently produced by different tumour cells.

Calcitonin immunoreactivity was present in 26 cases (46%) (Figure 2). Intensity of staining varied between different cases and also between the positive cells within each tumour. Focal groups of tumour cells showed stronger reactivity than those with the diffuse pattern. 15/57 cases showed bone formation. In seven of these there was diffuse positive staining for CL and small foci of cells with more intense staining were seen in some of them. The remaining six cases showed focal staining only. Two carcinoids with bone formation were negative for CL.

Positive staining for GS was demonstrated in 27 cases (47.4%). In the majority of these, GS immunoreactive cells predominated and were distributed evenly throughout the tumour (Figure 3). Only a small number of cases showed single groups of positive cells. SM immunoreactivity was seen in 19 cases (33%), where either intensely staining cells were a minority, being randomly scattered in the tumour or diffuse staining of most tumour cells was visible.

Sixteen tumours (28%) were positive for glucagon, the staining being focal in 6 cases and diffuse in 10. Glucagon was not found as the only peptide in any of the cases studied.

Of the four monoclonal antibodies used for the identification of ACTH only 2A3 and 3H9 demonstrated positive immunostaining and there was no difference in the pattern of staining using these two MAbs. Immunoreactivity was

**Table I** Immunohistologic staining in 57 bronchopulmonary carcinoid tumours

| | *Typical carcinoids* | | *Well differentiated neuroendocrine carcinomas* | |
|---|---|---|---|---|
| | *No. tumours with positive staining* | *Percentage* | *No. tumours with positive staining* | *Percentage* |
| Tumours studied | 30 | 53 | 27 | 47 |
| Positive | 29 | 97 | 18 | 67 |
| Negative | 1 | 3 | 9 | 33 |
| BN immunoreactivity | 20 | 67 | 10 | 37 |
| CL immunoreactivity | 17 | 57 | 9 | 33 |
| ACTH immunoreactivity | 17 | 57 | 7 | 26 |
| GS immunoreactivity | 15 | 50 | 12 | 44 |
| SM immunoreactivity | 13 | 43 | 6 | 22 |
| GLU immunoreactivity | 13 | 43 | 3 | 11 |

BN Bombesin, CL Calcitonin, GS Gastrin, SM Somatostatin, GLU Glucagon.

**Table II** The coexistence of peptides in bronchopulmonary carcinoid tumours

| | *Bombesin* | *Calcitonin* | *Gastrin* | *ACTH* | *Somatostatin* | *Glucagon* |
|---|---|---|---|---|---|---|
| Bombesin | (4) | 18 | 18 | 15 | 14 | 12 |
| Calcitonin | 18 | (3) | 14 | 13 | 12 | 13 |
| Gastrin | 18 | 14 | (3) | 13 | 9 | 12 |
| ACTH | 15 | 13 | 13 | (1) | 13 | 9 |
| Somatostatin | 14 | 12 | 9 | 13 | (3) | 11 |
| Glucagon | 12 | 13 | 12 | 9 | 11 | (–) |

[a]Figures in brackets are the number of cases positive for one peptide only.

**Table III** The degree of immunostaining and the distribution of immunoreactive cells

| *Peptides* | *Negative* | *1+F* | *F* | *2+D* | *F* | *3+D* | *F* | *4+D* |
|---|---|---|---|---|---|---|---|---|
| Bombesin | 27 | 5 | 3 | 1 | 10 | 9 | – | 2 |
| ACTH | 33 | 9 | 4 | – | 4 | 4 | – | 3 |
| Calcitonin | 31 | 5 | 4 | 2 | 6 | 9 | – | – |
| Gastrin | 30 | 5 | 3 | 2 | 5 | 12 | – | – |
| Somatostatin | 38 | 4 | 4 | – | 3 | 8 | – | – |
| Glucagon | 41 | 1 | 3 | – | 2 | 10 | – | – |

F focal pattern, D diffuse pattern 1+ is <20 cells positive, 2+ is 20–50 cells positive, 3+ is >50 cells positive, 4+ is when all tumour cells are positive.

present in 24 tumours (42%) (Figure 4), being focal in 17 and diffuse in 7. In this latter group three tumours showed staining of nearly every cell.

Normal bronchial tissue, containing sero-mucous glands and epithelium adjacent to the tumour, was present in sections from the majority of the central tumours studied. None of the cases showed positive staining for GS in the sero-mucous glands. However, antibodies to BN, CL, SM, GLU and ACTH demonstrated immunoreactivity in a variable number of serous and mucous glands (Figure 5). Peptide containing basal cells were seen in the epithelium in a small number of cases. The most commonly identified peptides were BN (Figure 6) and CL. A large number of GS and SM positive cells were seen in two cases. In one, both the tumour and bronchial epithelial cells were positive for GS, whereas in the other, where SM positive cells were present in bronchial epithelium, tumour cells were negative for this peptide. Basal peptide containing cells in the bronchial epithelium stained intensely but were less frequent than in glands. A relatively large number of peptide producing cells in metaplastic squamous epithelium was seen in one case where BN and CL were identified in the same areas of the section, but in apparently different cells. BN positive cells were more frequent than CL positive cells but tumour cells in this case were negative for both BN and CL. ACTH was identified in the adjacent epithelium in only one case.

### Discussion

Bronchial carcinoid tumours display immunoreactivity to a wide range of hormones and peptides and our results confirm this in that 47 of the 57 cases studied (83%) showed positivity for at least one of the peptides studies. Twenty two of the positive cases showed immunoreactivity for 3 to 5 peptides which indicated the capability of these tumours for synthesizing and storing more than one peptide simultaneously. This feature has been described previously in normal neuroendocrine cells (Larsson, 1980) and bronchial carcinoids (Yang *et al.*, 1983; Warren *et al.*, 1984). In a study of 25 bronchopulmonary carcinoids, Warren *et al.* (1984) have demonstrated up to eight hormones and their findings were supported by ultrastructural identification of a heterogeneous population of neurosecretory granules within single tumour cells. Multihormonal production has also been reported in small cell lung carcinoma cell lines which can secrete up to ten different peptides (Sorenson *et al.*, 1981).

The most frequently identified hormones in this study were bombesin (30 cases) followed by gastrin and calcitonin (27 and 26 cases) respectively. The finding of positive immunoreactivity for gastrin in a large number of our tumours is in contrast to three other studies which could not demonstrate the presence of gastrin (Yang *et al.*, 1983; Tamai *et al.*, 1983 & Martensson *et al.*, 1987). However, Warren *et al.*, (1984) found gastrin in 15/25 (60%) bronchial carcinoids studied. Although there is no clear explanation for these results it could relate to the ability of the antisera to recognise the various molecular forms such as Gastrin 1–17, 1–34, 1–14 and 1–5 (Walsh & Grossman, 1975).

The results of immunostaining for bombesin are in agreement with previous reports. We observed immunoreactivity in 67% of typical carcinoids and 37% of atypical carcinoids in comparison with the study by Hamid *et al.* (1987) which showed moderately strong immunostaining in 35% of benign carcinoids and 22% of atypical carcinoids. The difference in the prevalence could relate to a difference in the specificity of the antisera, but this cannot be confirmed because the relevant specificity is not detailed in their study, and it may anyway relate to a difference in the ability of the antisera to recognise their epitopes in the precursor molecule. The relative immunoreactivity for bombesin was confirmed in the series of Hamid *et al.* (1987) by radioimmunoassay of tumour extracts from paraffin blocks suggesting that the immunostaining is a reliable approach for examining the expression of bombesin in bronchopulmonary carcinoids. Since high levels of bombesin have been reported in cell lines from small cell lung cancer (Moody *et al.*, 1981; Wood *et al.*, 1981; Sorenson *et al.*, 1982) and in pulmonary carcinoid tumours (Bostwick *et al.*, 1984; Martensson *et al.*, 1987).

Calcitonin, a 29 amino acid peptide, is predominantly found in thyroid C-cells and their derivative tumour, medullary carcinoma. In this study calcitonin was found in 46% of bronchopulmonary carcinoids. This is more frequent than recent reports where Warren *et al.* (1984) showed calcitonin immunoreactivity in 8% of bronchial carcinoids and Martensson *et al.* (1987) in 22% whereas Yang *et al.* (1983) were unable to demonstrate reactivity in seven tumours. Eighteen of the 26 cases with immunoreactivity for calcitonin in the present study were also positive for bombesin. The frequent demonstration of bombesin and calcitonin in our series parallels previous studies of normal endocrine cells of the bronchopulmonary tree (Cutz *et al.*, 1981) and bronchopulmonary carcinoids (Warren *et al.*, 1984), and in normal and neoplastic thyroid C-cells. The finding of bombesin and calcitonin in separate cells in this study could indicate that the hormones are not co-secreted. The other frequent combinations of peptides in our series were gastrin and bombesin (18 cases), bombesin and ACTH (15 cases) and gastrin and calcitonin (14 cases).

ACTH was absent in the cases described by Warren *et al.* (1984) but was seem in 28% of tumours in the study of Martensson *et al.* (1987). In common with these authors the present study shows ACTH was the second commonest pattern of immunostaining shown in bronchopulmonary carcinoids. The differences between these studies could well be due to the antibodies used, Warren *et al.* (1984) used Porcine anti-ACTH 1–39 antibodies at a dilution of 1/400, Martensson *et al.* (1987) used ACTH antisera at a dilution of 1/160 obtained from MILAB, Malmo, Sweden.

There is a suggestion that the production of calcitonin in bronchopulmonary carcinoid tumours may induce ossification of bronchial cartilage as well as bone formation in the tumour (Cooney *et al.*, 1979). Fifteen (26%) of the tumours in our study showed bone formation, 13 of which were positive for calcitonin. However, a further 12 positive cases showed no bone suggesting that no such correlation exists. It is not logical to expect calcitonin to induce ossification since bone is rare in medullary carcinoma of thyroid, which has high levels of calcitonin. Calcification and the presence of psammoma bodies have been reported in this tumour (Williams *et al.*, 1966).

Well differentiated neuroendocrine carcinomas ('atypical or malignant carcinoids') did not stain positively as frequently as typical carcinoids. One third of the neuroendocrine carcinomas were negative for hormones as opposed to 3% of the typical carcinoids. Somatostatin and glucagon were found least and bombesin was found most often. One reason for lack of staining could be that the less well differentiated neuroendocrine carcinomas produce either smaller peptides or smaller quantities of peptides.

Finally it may be asked what is the clinical relevance of the peptide production demonstrated in this study. Only three cases showed clinical effects of hormone release; two

Figure 1

Figure 2

Figure 3

Figure 4

Figure 5

Figure 6

**Figure 1** Typical carcinoid staining strongly to bombesin (Immunoperoxidase × 250).

**Figure 2** Atypical carcinoid tumour with strong positive staining for calcitonin (Immunoperoxidase × 250).

**Figure 3** Typical carcinoid with almost all tumour cells staining for gastrin (Immunoperoxidase × 400).

**Figure 4** Atypical carcinoid. Focal positive staining for ACTH with MAb 3H9 (Immunoperoxidase × 250).

**Figure 5** Immunoreactivity to calcitonin at bronchial resection margin in mucous gland (Immunoperoxidase × 250).

**Figure 6** Epithelial cells staining for bombesin in the epithelium of resection margin of a bronchial carcinoid. (Immunoperoxidase × 400).

had the carcinoid syndrome and one showed features of ectopic growth hormone production (Shalet et al., 1979). However, this study covers a period of 25 years and it is likely that endocrine manifestations were missed in the early years. Despite this it still appears there is a disparity between the clinical manifestations of bronchial carcinoid tumours and the large number of cases showing immunoreactivity. One explanation is that the pulmonary endothelial cells can detoxicate or degrade hormones so they do not reach the systemic circulation.

It should finally be noted that non-small cell carcinoma tumours can secrete hormones. Mooi et al. (1988) have shown in 11 cases, classified as large or squamous cell carcinoma, positive staining for neuron specific enolase, protein gene product 9.5 and C-terminal peptide of human pro-bombesin and chromogranin.

Dr Al-Saffar had a Post Doctoral Visiting Fellowship at the Paterson Institute, Christie Hospital, Manchester. Dr A. White is funded by the North West Regional Health Authority.

### References

BOSTWICK, D.G., ROTH, K.A., EVANS, C.J., BARCHAS, J.D. & BENSCH, K.G. (1984). Gastrin releasing peptide, a mamalian analog of bombesin, is present in human neuroendocrine lung tumours. *Am. J. Pathol.*, **117**, 195.

COONEY, T., SWEENEY, E. & LUKE, D. (1979). Pulmonary carcinoid tumours: A comparative regional study. *J. Clin. Pathol.*, **32**, 1100.

CUTZ, E., CHAN, W. & TRACK, N.S. (1981). Bombesin, Calcitonin and Leuenkephalin immunoreactivity in endocrine cells of human lung. *Experientia*, **37**, 765.

HAMID, Q.A., ADDIS, B.J., SPRINGALL, D.R. & 4 others (1987). Expression of the C-terminal peptide of human pro-bombesin in 361 lung endocrine carcinomas, a reliable marker and possible prognostic indicator for small cell carcinoma. *Virchows Arch. A.*, **411**, 185.

HASLETON, P.S., GOMM, S., BLAIR, V. & THATCHER, N. (1986). Pulmonary carcinoid tumours: A clinicopathological study of 35 cases. *Br. J. Cancer*, **54**, 963.

LARSSON, L.I. (1980). The possible existence of multiple endocrine, paracrine and neurocrine messengers in secretory cell system. *Invest. Cell Pathol.*, **3**, 73.

MARTENSSON, H., BOLTCHER, G., HAMBRAEUS, G., SUNDLER, G., WILLEN, H. & NOBIN, A. (1987). Bronchial carcinoids: An analysis of 91 cases. *World J. Surg.*, **11**, 356.

MOODY, T.W., PERT, C.B., GAZDER, A.F., CARNEY, D.N. & MINNA, J.D. (1981). High levels of intracellular bombesin characterize human small cell lung carcinoma. *Science*, **214**, 1246.

MOOI, W.J., DEWART, T., SPRINGALL, D.R., POLAK, J. & ADDIS, B.J. (1980). Non-small cell lung carcinomas with neuroendocrine features. A light microscopic immunohistochemical and ultrastructural study of 11 cases. *Histopathol.*, **13**, 329.

PRICE, J., NICUWENHUIJZEN KRUSEMAN, A.C., DONIACH, I., HOWLETT, T.A., BESSER, G.M. & REES, L.H. (1983). Bombesin-like peptides in human endocrine tumours: Quantitation, biochemical characterisation and secretion. *J. Clin. Endocrinol. Metabol.*, **60**, 1097.

SHALET, S.M., BEARDWELL, C.G., MACFARLANE, I.M. & 4 others (1979). Acromegaly due to production of a growth hormone releasing factor by a bronchial carcinoid tumour. *Clin. Endocrinol.*, **10**, 61.

SORENSON, G.D., PETTENGIL, O.S., BRINCK-JOHNSON, T., CATE, C.G. & MAURER, L.H. (1981). Hormone production by cultures of small cell carcinoma of the lung. *Cancer*, **47**, 1289.

SORENSON, G.D., BLOOM, S.R., GHATEI, M.A., DELPRETE. S.A., CATE, C.G. & PETTENGILL, O.S. (1982). Bombesin production by human small cell carcinoma of the lung. *Regul Peptides*, **4**, 56.

STERNBERGER, L.A. (1979). *Immunocytochemistry* 2nd Edition. Wiley and Sons, New York.

TAMAI, S., KAMEYA, T., YAMAGUCHI, K. & 5 others (1983). Peripheral lung carcinoid tumour producing predominantly gastrin-releasing peptide (GRP). Morphologic and hormonal studies. *Cancer*, **52**, 273.

WALSH, J.H. & GROSSMAN, M.I. (1975). Gastrin. *N. Engl. J. Med.*, **292**, 1324.

WARREN, W.H., MEMOLI, V.A. & GOULD, V.E. (1984). Immunohistochemical and Ultrastructural analysis of bronchopulmonary neuroendocrine neoplasms. 1. Carcinoids. *Ultrast. Pathol.*, **6**, 15.

WHITE, A., GRAY, C. & RATCLIFFE, J.G. (1985). Characterisation of monoclonal antibodies to adrenocorticotrophin *J. Immunol. Methods*, **79**, 185.

WILLIAMS, E.D., BROWN, C.L. & DONIACH, I. (1966). Pathological and clinical findings in a series of 67 cases of medullary carcinoma of the thyroid. *J. Clin Pathol.*, **19**, 103.

WOOD, S.M., WOOD, J.R., GHATEI, M.A., LEELL, C., SHAUGHNESSY, D. & BLOOM, S.R. (1981). Bombesin, Somatostatin and neurotensin-like immunoreactivity in bronchial carcinoma. *J. Clin. Endocrinol.*, **53**, 1310.

YANG, K., ULICH, T., TAYLOR, I., CHENG, L. & LEWIN, K.J. (1983). Pulmonary carcinoids. Immunohistochemical demonstration of brain-gut peptides. *Cancer*, **52**, 819.